# Protective Effects of Polysaccharides from *Pyropia suborbiculata* Against UVB-Induced Photodamage in HaCaT Cells

**DOI:** 10.3390/foods15081292

**Published:** 2026-04-09

**Authors:** Kaiyue Chen, Hongchang Ding, Jiawei Zhong, Qinwen Zhou, Yujia Li, Long Zhang, Quancai Sun, Ye Peng, Wenhui Wu, Xichang Wang, Wanqiang Wu

**Affiliations:** 1 College of Food Science and Technology, Shanghai Ocean University, Shanghai 201306, China; 18638709512@163.com (K.C.); jiaweizhong1215@163.com (J.Z.); 17714586689@163.com (Q.Z.); lyj20020127@163.com (Y.L.); l-zhang@shou.edu.cn (L.Z.); 2 Key Laboratory of Exploration and Utilization of Aquatic Genetic Resources, Ministry of Education, Shanghai Ocean University, Shanghai 201306, China; hcding@shou.edu.cn; 3 Shanghai Engineering Research Center of Aquaculture, Shanghai Ocean University, Shanghai 201306, China; 4 Shanghai Engineering Research Center of Aquatic-Product Processing and Preservation, Shanghai 201306, China; 5 Faculty of Medicine, Macau University of Science and Technology, Taipa, Macao, China; qcsun@must.edu.mo (Q.S.); pengye@must.edu.mo (Y.P.); 6 Marine Biomedical Science and Technology Innovation Platform of Lin-Gang Special Area, Shanghai 201306, China; whwu@shou.edu.cn; 7 Department of Marine Pharmacology, College of Food Science and Technology, Shanghai Ocean University, Shanghai 201306, China; 8 Putuo Sub-Center of International Joint Research Center for Marine Biological Sciences, Zhoushan 316104, China; 9 International Research Center for Food and Health, College of Food Science and Technology, Shanghai Ocean University, Shanghai 201306, China

**Keywords:** *Porphyra suborbiculata* PS-M4, polysaccharide, extraction and purification, structural, anti-photodamage, HaCaT keratinocytes

## Abstract

*Porphyra suborbiculata* exhibits strong heat tolerance and has considerable commercial potential under rising sea temperatures; however, its bioactive components remain insufficiently explored. In this study, a heat-tolerant new strain of *P. suborbiculata* (PS-M4), cultivated by the College of Fisheries, was used as the experimental material. Polysaccharides were extracted using an ultrasound-assisted composite enzymatic method, and extraction conditions were optimized through single-factor experiments and response surface methodology, yielding a maximum extraction yield of 12.45 ± 0.09%. Crude polysaccharides were further purified using a purification apparatus, yielding two fractions, designated PSP-I and PSP-II. Preliminary structural characterization showed that PSP-I possessed a weight-average molecular weight (Mw) of 26.149 kDa, a number-average molecular weight (Mn) of 11.267 kDa, and a polydispersity index of 2.321. Monosaccharide composition analysis indicated that PSP-I was predominantly composed of galactose. Fourier transform infrared spectroscopy (FT-IR) revealed typical polysaccharide functional groups, and scanning electron microscopy (SEM) analysis revealed a porous lamellar morphology. In vitro cell-based assays demonstrated that PSP-I significantly alleviated ultraviolet B (UVB)-induced damage in HaCaT cells by reducing intracellular reactive oxygen species (ROS) levels, enhancing antioxidant enzyme activities, inhibiting apoptosis, and downregulating the expression of matrix metalloproteinases (MMPs). These results suggest that PSP-I has potential as a functional ingredient for mitigating UVB-induced skin damage.

## 1. Introduction

Skin damage induced by ultraviolet (UV) radiation has become an increasingly important global public health concern, driven by increasing environmental pollution and stratospheric ozone depletion. Solar ultraviolet radiation in sunlight can be classified into three types according to wavelength: UVA (315–400 nm), UVB (280–315 nm), and UVC (100–280 nm). While UVC is almost completely absorbed by the atmospheric ozone layer, approximately 2–5% of UVB reaches the Earth’s surface and penetrates the epidermal layer, making it a primary contributor to skin photodamage [1,2,3]. UVB exposure triggers excessive production of reactive oxygen species (ROS) in skin cells, disrupting cellular redox homeostasis and initiating oxidative stress. This process is widely recognized as a key mechanism underlying UV-induced skin damage [4,5].

At the molecular level, UVB-induced oxidative stress activates the mitogen-activated protein kinase (MAPK) signaling pathway, which in turn upregulates the transcription factor activator protein-1 (AP-1) and promotes excessive expression of matrix metalloproteinases (MMPs). These enzymes degrade collagen and elastin in the dermis, compromising the structural integrity of the skin and contributing to wrinkle formation and loss of elasticity [4,6,7]. In addition, UVB radiation can directly induce deoxyribonucleic acid (DNA) damage in skin cells through the formation of cyclobutane pyrimidine dimers, which are closely associated with the development of both melanoma and non-melanoma skin cancers [8]. These molecular alterations ultimately manifest as visible skin damage, including sunburn, tanning, photoaging, and skin carcinogenesis [9]. With growing awareness of the harmful effects of UV radiation, the development of effective and safe strategies to protect against UV-induced skin damage has become a major focus in skin health research.

Currently, topical antioxidants such as vitamins C, E, and polyphenols are commonly used for photoprotection; however, their application is often limited by poor stability, low skin permeability, and susceptibility to degradation by air or light [10]. In addition, most active ingredients in commercial sunscreens function as UVB absorbers and are mainly inorganic or organic compounds, such as titanium dioxide and hydroxybenzophenone, which raise ongoing concerns regarding long-term safety [11]. In contrast, natural active ingredients have attracted increasing attention due to their favorable safety profiles, low irritation, and good biocompatibility, making them a growing trend in skincare product development [12]. To date, research on anti-photoaging agents has mainly focused on medicinal and edible plants, such as *Ganoderma lucidum* and *Lycium barbarum*, whereas relatively limited attention has been paid to natural products derived from food sources. Notably, marine organisms are increasingly recognized as an important reservoir of bioactive compounds, owing to their unique living environments and metabolic characteristics [13].

Marine-derived natural products have already achieved broad commercial application. For instance, docosahexaenoic acid (DHA) derived from the marine protist *Schizochytrium* is widely used in infant formula, functional foods, and dietary supplements, supported by its proven bioavailability and cardiovascular and neuroprotective benefits [14]. In the aquaculture industry, bioactive molecules from algae, crustaceans, and fish—including astaxanthin, carotenoids, chitosan, fucoidan, lectins, and polyunsaturated fatty acids (PUFAs)—are among the most commonly applied feed additives [15]. In the cosmetics sector, astaxanthin from *Haematococcus pluvialis* has been incorporated into skincare products due to its potent antioxidant, anti-aging, and photoprotective properties [16].

Algal polysaccharides are among the most abundant bioactive components in marine algae and are polyhydroxy polymers composed of more than 20 monosaccharides linked by glycosidic bonds. Accounting for more than 76% of algal dry weight, these polysaccharides exhibit a wide range of biological activities, including antioxidant, hypolipidemic, antitumor, and anti-inflammatory effects [17]. For example, Park et al. [18] demonstrated that polysaccharides extracted from *Codium fragile* act as immunostimulatory agents by activating natural killer cells, suggesting their potential as therapeutic agents for cancer treatment. Yan et al. [19] reported that two polysaccharides isolated from *Codium cylindricum* using ultrasonic extraction showed enhanced antioxidant activity after molecular weight reduction. In addition, Oh et al. [20] confirmed the anti-obesity effects of *Codium fragile* polysaccharides by suppressing adipocyte differentiation. Despite these findings, algae are still used primarily as foods or seasonings, and research has largely focused on basic biological activities such as antioxidant and anti-inflammatory effects. However, photoaging involves complex biological processes, including oxidative stress, inflammation, and apoptosis. Therefore, further investigation into the photoprotective and anti-photoaging activities of algal polysaccharides is needed to promote their high-value utilization [13]. In particular, systematic studies on the composition, structure, and bioactivity of polysaccharides from specific algae remain limited, highlighting the considerable potential of marine polysaccharides as functional bioactive resources.

*Porphyra suborbiculata* is a thermophilic seaweed belonging to the genus *Porphyra* and is considered a relatively rare species. It occurs along the Chinese coastline from 22° N to 38° N latitude and is rich in nutrients and bioactive compounds [21]. Previous studies have shown that, compared with commonly cultivated species such as *Porphyra yezoensis* and *Porphyra haitanensis*, *P. suborbiculata* shows superior heat tolerance. In the context of global warming and rising sea temperatures, this characteristic suggests considerable potential for future commercial development. However, systematic studies on its chemical composition and functional properties remain limited. To overcome the limitations of wild round laver strains, which typically exhibit small, round, or kidney-shaped thalli and are not suited to large-scale cultivation, Professor Ding Hongchang’s group at the College of Fisheries, Shanghai Ocean University, developed improved strains through artificial mutagenesis. Elongated mutants (PS-5) were first obtained from wild-type strains, followed by further induction using ^60^Co-γ irradiation. A fast-growing strain, designated PS-M4, was subsequently selected based on growth rate and blade morphology [22]. Marine field trials demonstrated that PS-M4 showed significant improvements in blade growth, quality, and chlamydospore release, meeting production requirements in both morphology and yield. These results indicate that PS-M4 is a promising candidate for large-scale cultivation with enhanced commercial value [21].

The present study focuses on the novel PS-M4 strain of *Porphyra suborbiculata*. An ultrasonic-assisted composite enzymatic method was employed to optimize the extraction of polysaccharides from this strain. The in vitro antioxidant activity of the crude polysaccharides was evaluated, and the crude polysaccharides were further purified. The structure of the obtained polysaccharides and their protective effects against UVB-induced skin damage were preliminarily investigated. The aims of this study were threefold: (i) to provide scientific support for the high-value utilization of marine biological resources; (ii) to promote the potential commercialization of the PS-M4 strain; and (iii) to offer new insights into the prevention and amelioration of UVB-induced skin damage.

## 2. Materials and Methods

### 2.1. Materials and Reagents

*Porphyra suborbiculata* (PS-M4) specimens were collected from Ningde, Fujian Province, China, in November 2024. After thorough washing and drying, the samples were stored in vacuum-sealed containers at 4 °C. DEAE Sepharose Fast Flow and Sephadex G-75 were obtained from Taidu Biotechnology Co., Ltd. (Suzhou, Jiangsu, China). HaCaT cells (RRID: CVCL_0038) were obtained from Haixing Biotechnology Co., Ltd. (Suzhou, China). Dulbecco’s modified Eagle’s medium (DMEM) was purchased from Shenzhen Comma Biotechnology Co., Ltd. (Shenzhen, China). Cell Counting Kit-8 (CCK-8) was obtained from Biosharp Biotechnology Co., Ltd. (Hefei, Anhui, China). Enzyme-linked immunosorbent assay (ELISA) kits for matrix metalloproteinase-1 (MMP-1), matrix metalloproteinase-3 (MMP-3), and matrix metalloproteinase-9 (MMP-9) were purchased from Jiangsu AidiSheng Biotechnology Co., Ltd. (Yancheng, Jiangsu, China). Kits for the measurement of reactive oxygen species (ROS), superoxide dismutase (SOD), and glutathione peroxidase (GSH-Px) were obtained from Shanghai Beyotime Biotechnology Co., Ltd. (Shanghai, China). Hoechst 33342 staining solution was purchased from Shanghai Yuanye Biotechnology Co., Ltd. (Shanghai, China). All other reagents were of analytical grade.

### 2.2. Preparation of Polysaccharides

#### 2.2.1. Single-Factor Experimental Design

Polysaccharides in algal cells are primarily found in the cell wall, which is mainly composed of cellulose, hemicellulose, and pectin [23]. Accordingly, a synergistic enzymatic hydrolysis approach using cellulase and pectinase, combined with ultrasound-assisted cavitation, was employed to disrupt the cell wall structure and facilitate polysaccharide release. Single-factor experiments were performed to evaluate the effects of the following parameters on extraction yield: solid-to-liquid ratio (1:10, 1:20, 1:30, 1:40, and 1:50 g/mL), cellulase dosage (0.5%, 1.0%, 1.5%, 2.0%, and 2.5%), pectinase dosage (0.5%, 1.0%, 1.5%, 2.0%, and 2.5%), extraction temperature (40, 50, 60, 70, and 80 °C), extraction time (30, 60, 90, 120, and 150 min), and ultrasonic power (120, 150, 180, 210, and 240 W). The polysaccharide yield was calculated as follows:

(1) $$\mathrm{Yield}\;\left(\%\right)=\frac{C\times V\times D}{M}\times 100\%$$

where C indicates the polysaccharide concentration determined from the glucose standard curve (mg/mL); V represents the volume of the polysaccharide solution (mL); D indicates the dilution factor; M represents the mass of dried *P. suborbiculata* powder (mg).

#### 2.2.2. Box–Behnken Design

Based on the results of the single-factor experiments, the variables with significant effects were selected as independent factors: ultrasonic power (A), extraction temperature (B), cellulase addition (C), and pectinase addition (D), while polysaccharide extraction yield was used as the response variable (Y). A Box–Behnken design (BBD) with four factors and three levels was performed using Design-Expert 13.0. The experimental design consisted of 29 runs, including five center points, to determine the optimal extraction conditions for polysaccharide yield.

#### 2.2.3. Extraction of Crude Polysaccharides

The dried laver was ground, passed through a 40-mesh sieve, and extracted three times with anhydrous ethanol to remove pigments. The de-pigmented powder was suspended in citric acid–sodium dihydrogen phosphate buffer (pH 5.0), followed by enzymatic hydrolysis using a compound enzyme preparation (cellulase and pectinase) under ultrasonic treatment. The reaction was terminated by boiling for 10 min, and the mixture was then centrifuged at 10,000 rpm for 30 min. The supernatant was precipitated with a threefold volume of anhydrous ethanol at 4 °C overnight and then centrifuged to obtain crude polysaccharides. Protein removal was performed using the trichloroacetic acid (TCA) method: an appropriate amount of crude polysaccharide was dissolved in water, followed by the dropwise addition of 20% TCA solution to a final concentration of 4%. The mixture was thoroughly mixed and kept at 4 °C overnight. After centrifugation, the supernatant was collected, and the pH was adjusted to 7.0. The sample was dialyzed using a membrane with a molecular weight cut-off (MWCO) of 3500 Da for 72 h and then freeze-dried to obtain the preliminarily purified polysaccharide (PSP) [24]. Protein removal efficiency was evaluated by UV scanning in the range of 200–600 nm.

#### 2.2.4. In Vitro Antioxidant Activity of PSP

To preliminarily evaluate the in vitro antioxidant activity of the crude polysaccharide PSP, four assays were conducted: 2,2-diphenyl-1-picrylhydrazyl (DPPH) radical-scavenging activity, superoxide anion-scavenging activity, total reducing power, and Fe^2+^-chelating activity. These were conducted following the protocols outlined by Wang et al. [25], Tian et al. [23], Thambiraj et al. [26] and Zhu et al. [27], with minor modifications. Solutions of the polysaccharides at varying concentrations (1, 2, 3, 4, and 5 mg/mL) were prepared, and vitamin C (Vc) and ethylenediaminetetraacetic acid (EDTA) were used as positive controls. All tests were performed in triplicate to ensure data reliability.

#### 2.2.5. Purification of Crude Polysaccharides

Crude PSP (150 mg) was dissolved in 40 mL of Tris–HCl buffer and subjected to anion-exchange chromatography on a DEAE Sepharose Fast Flow column (20 cm × 1.6 cm) using a purification system (Suzhou Taidu Biotechnology Co., Ltd., Suzhou, China). Elution was performed sequentially with Tris–HCl buffer containing 0, 0.1, 0.3, 0.5, 0.7, and 0.9 M NaCl at a flow rate of 3 mL/min. Polysaccharide fractions were monitored at 490 nm using the phenol–sulfuric acid method. Based on peak area, the fractions eluted with 0 and 0.5 M NaCl were collected, dialyzed (MWCO 3500 Da), and freeze-dried for further purification. The collected fractions were further purified on a Sephadex G-75 column (40 cm × 2.6 cm; Suzhou Taidu Biotechnology Co., Ltd., Suzhou, China) and eluted with deionized water [28]. The eluate corresponding to the main peak was collected, followed by dialysis using a membrane with a molecular weight cut-off (MWCO) of 3500 Da and freeze-drying, yielding the purified polysaccharides designated PSP-I and PSP-II.

### 2.3. Compositional Characterization

#### 2.3.1. Molecular Weight Determination

Molecular weight was determined by size-exclusion chromatography coupled with multi-angle laser light scattering and differential refractive index detection (SEC–MALS–RI) [29]. Samples were dissolved in 0.1 M NaNO_3_ containing 0.02% (*w*/*w*) NaN_3_ to a final concentration of 1 mg/mL and filtered through a 0.45 μm membrane before analysis. The SEC system consisted of an UltiMate 3000 (Thermo Fisher Scientific, Waltham, MA, USA) equipped with an Optilab T-rEX differential refractive index detector and a DAWN HELEOS II MALS detector (Wyatt Technology, Santa Barbara, CA, USA). Separation was performed on Ohpak SB-805 HQ (300 × 8 mm) and Ohpak SB-803 HQ (300 × 8 mm) columns connected in series at 45 °C. The mobile phase was 0.1 M NaNO_3_ containing 0.02% NaN_3_, with a flow rate of 0.6 mL/min, and the injection volume was 100 μL.

#### 2.3.2. Monosaccharide Composition Analysis

Fifteen monosaccharide standards, namely fucose, rhamnose, arabinose, galactose, glucose, xylose, galactosamine, glucosamine, mannose, fructose, ribose, galacturonic acid, glucuronic acid, mannuronic acid, and guluronic acid, were used for monosaccharide identification and the construction of standard curves. Polysaccharide samples were hydrolyzed with 2 M trifluoroacetic acid (TFA) at 121 °C for 2 h. After nitrogen purging and complete drying, residual TFA was removed by repeated washing with methanol (2–3 times), followed by drying. The hydrolysate was dissolved in ultrapure water, filtered through a 0.22 μm membrane, and transferred to chromatography vials for analysis. Monosaccharide composition was analyzed by high-performance anion-exchange chromatography with pulsed amperometric detection (HPAEC–PAD) using an ICS 5000+ system (Thermo Fisher Scientific, Waltham, MA, USA) equipped with a Dionex™ CarboPac™ PA-20 column (3 × 150 mm, 10 μm). A 5 μL aliquot was injected. The mobile phases were water (A), 0.1 M NaOH (B), and 0.1 M NaOH containing 0.2 M sodium acetate (C). The flow rate was 0.5 mL/min, and the column temperature was maintained at 30 °C. The elution program was as follows: 0–26 min, 95% A and 5% B; 26–42 min, 85% A, 5% B, and 10% C; 42–42.1 min, 60% A and 40% C; 42.1–52 min, 60% A and 40% B; and 52–60 min, 95% A and 5% B. The total run time was 60 min. Monosaccharides were identified by comparing their retention times with those of authentic standards [30].

#### 2.3.3. Fourier Transform Infrared Spectroscopy (FT-IR) Analysis

FT-IR spectra of PSP-I were recorded using a Nicolet iS10 spectrometer (Thermo Fisher Scientific, Waltham, MA, USA) equipped with a deuterated triglycine sulfate (DTGS) detector. The dried sample was ground with KBr powder and pressed into a pellet. Spectra were collected over the range of 4000–600 cm^−1^ at a resolution of 4 cm^−1^ with 32 scans.

#### 2.3.4. Scanning Electron Microscopy (SEM) Analysis

The dried PSP-I sample (5 mg) was mounted onto a metal stub using double-sided conductive tape and sputter-coated with gold for 40 s. The morphology was observed using a Hitachi SU5000 field-emission scanning electron microscope (Hitachi, Japan) equipped with a secondary electron detector at an accelerating voltage of 5.0 kV. Images were recorded at magnifications of ×200, ×400, ×600, and ×1500.

### 2.4. UVB Photoprotection Activity Evaluation

#### 2.4.1. Cell Culture

HaCaT cells were cultured in DMEM supplemented with 10% (*v*/*v*) fetal bovine serum (FBS) and 1% (*v*/*v*) penicillin–streptomycin at 37 °C in a humidified incubator containing 5% CO_2_. Cells at passages 4–6 were used for subsequent experiments.

#### 2.4.2. Cytotoxicity of PSP-I and UVB Radiation in HaCaT Cells

When HaCaT cells reached 80–90% confluence, they were seeded into 96-well plates at a density of 1 × 10^4^ cells per well. After 24 h of attachment, the cells were used for cytotoxicity evaluation and UVB damage model establishment.

PSP-I was prepared as a 5 mg/mL stock solution. After appropriate dilution with culture medium, it was added to the wells to achieve final concentrations of 1000, 800, 400, 200, and 100 μg/mL, with three replicate wells for each concentration. The cells were then incubated for 24 h. A cell control group (without PSP-I) and a blank group (without cells) were included.

UVB irradiation was performed using a Philips 9 W UVB lamp (311 nm, Philips PL-S 9W/01, Eindhoven, The Netherlands) positioned 10 cm above the cells. Cells were divided into a control group (non-irradiated) and experimental groups exposed to increasing UVB doses (0–90 mJ/cm^2^), with a blank group included. Before irradiation, the culture medium was removed, cells were washed once with phosphate-buffered saline (PBS), and 50 μL of PBS was added to each well to cover the cells during irradiation [6,31]. After irradiation, PBS was aspirated, and 100 μL of complete medium was added to each well, followed by further incubation for 24 h. The optimal radiation dose was selected based on cell viability assessment.

Cell viability was determined using the CCK-8 assay, with the control group defined as 100% viability. Cell viability was calculated as follows:

(2) $$\mathrm{Cell}\;\mathrm{viability}\;\left(\%\right)=\frac{A_{\exp}-A_{\mathrm{blank}}}{A_{\mathrm{ctrl}}-A_{\mathrm{blank}}}\times 100\%$$

where A_exp_ indicates the absorbance of the experimental group; A_blank_ indicates the absorbance of the blank group (wells without cells); A_ctrl_ represents the absorbance of the control group (untreated cells).

#### 2.4.3. Protective Effect of PSP-I on Cell Viability

When HaCaT cells reached 80–90% confluence, they were seeded into 96-well plates at a density of 1 × 10^4^ cells per well and incubated at 37 °C in a humidified atmosphere containing 5% CO_2_ atmosphere for 12 h to allow attachment. Polysaccharides were diluted to final concentrations of 800, 400, and 200 μg/mL and added to the cells, while the positive control group was treated with sodium hyaluronate (Mw 300 kDa, designated HA30) at 200 μg/mL. After 6 h of pretreatment, the model, positive control, and experimental groups were exposed to UVB irradiation at a dose of 40 mJ/cm^2^ to establish the injury model, whereas the control group was not irradiated. Cells were then incubated with the corresponding treatments for an additional 24 h, with three replicate wells per group. Cell viability was determined using the CCK-8 assay, with the control group defined as 100% viability. The cell viability was calculated using the same formula as Equation (2).

#### 2.4.4. Determination of Reactive Oxygen Species (ROS), Superoxide Dismutase (SOD), and Glutathione Peroxidase (GSH-Px) Levels

HaCaT cells were seeded into 6-well plates at a density of 3 × 10^6^ cells per well and incubated at 37 °C in a 5% CO_2_ atmosphere for 12 h to allow attachment (reaching 80–90% confluence). Subsequent treatments, including polysaccharide incubation and UVB irradiation, were performed as described in Section 2.4.3. After irradiation, cells were further cultured for 24 h.

For intracellular ROS detection, the culture medium was removed, and cells were washed once with PBS. A 1000-fold diluted 2′,7′-dichlorodihydrofluorescein diacetate (DCFH-DA) probe (Beyotime, Shanghai, China) was added to each well and incubated for 20 min at 37 °C. The probe solution was then removed, and cells were washed three times with PBS to eliminate excess probe. Fluorescence images were captured using a Mshot MF31 fluorescence microscope (Guangzhou, China) at excitation and emission wavelengths of 488 nm and 525 nm, respectively [3].

SOD activity was determined using a WST-8–based colorimetric assay. Total GSH-Px activity was measured using the NADPH-based method. All measurements were performed according to the manufacturers’ instructions.

#### 2.4.5. Assessment of Apoptosis

Cell seeding, polysaccharide treatment, and UVB irradiation were performed as described in Section 2.4.4. After 24 h of post-irradiation culture, the supernatant was discarded, and cells were washed once with PBS. Hoechst 33342 staining solution was added to each well, followed by incubation for 15 min at 37 °C in the dark [32,33]. Cells were then washed three times with PBS, and apoptotic body formation was observed using a fluorescence microscope.

#### 2.4.6. Determination of MMP Levels

For MMP analysis, cells were treated as described in Section 2.4.4 and cultured for 24 h after UVB exposure. The culture supernatant was collected and centrifuged at 3000 rpm for 20 min at 4 °C to remove debris. The levels of matrix metalloproteinase-1 (MMP-1), MMP-3, and MMP-9 were determined using double-antibody sandwich enzyme-linked immunosorbent assay (ELISA) kits (Jiangsu Aidison Biotechnology Co., Ltd., Yancheng, China), following the manufacturers’ instructions. The detection ranges were 5–240 ng/mL for MMP-1, 1–320 ng/mL for MMP-3, and 0.1–48 ng/mL for MMP-9, with sensitivities of <5 ng/mL, <1 ng/mL, and <0.1 ng/mL, respectively. The levels of MMP-1, MMP-3, and MMP-9 were expressed as percentages of the control group, which was set to 100%.

### 2.5. Statistical Analysis

Response surface methodology (RSM) optimization was performed using Design-Expert 13.0 software (Stat-Ease Inc., Minneapolis, MN, USA). Data analysis for cell-based assays was conducted using IBM SPSS Statistics 26 software with Waller-Duncan test for multiple comparisons. All experiments were repeated three times, and the data are presented as mean ± standard deviation (SD). A value of *p* < 0.05 was considered statistically significant. Graphical processing was carried out using Origin 2021 software.

## 3. Results

### 3.1. Optimization of Polysaccharide Extraction

Through single-factor experiments, the factors significantly affecting the extraction yield were identified as ultrasonic power, extraction temperature, cellulase dosage, and pectinase dosage. A four-factor, three-level response surface design was then employed to optimize these parameters. The optimal conditions were determined as follows: ultrasonic power of 180 W, extraction temperature of 60 °C, cellulase dosage of 1.7%, pectinase dosage of 1.0%, extraction time of 90 min, and solid-to-liquid ratio of 1:30, yielding a polysaccharide extraction yield of 12.45 ± 0.09%.

#### 3.1.1. Single-Factor Experiments on Polysaccharide Extraction

**Figure 1:**
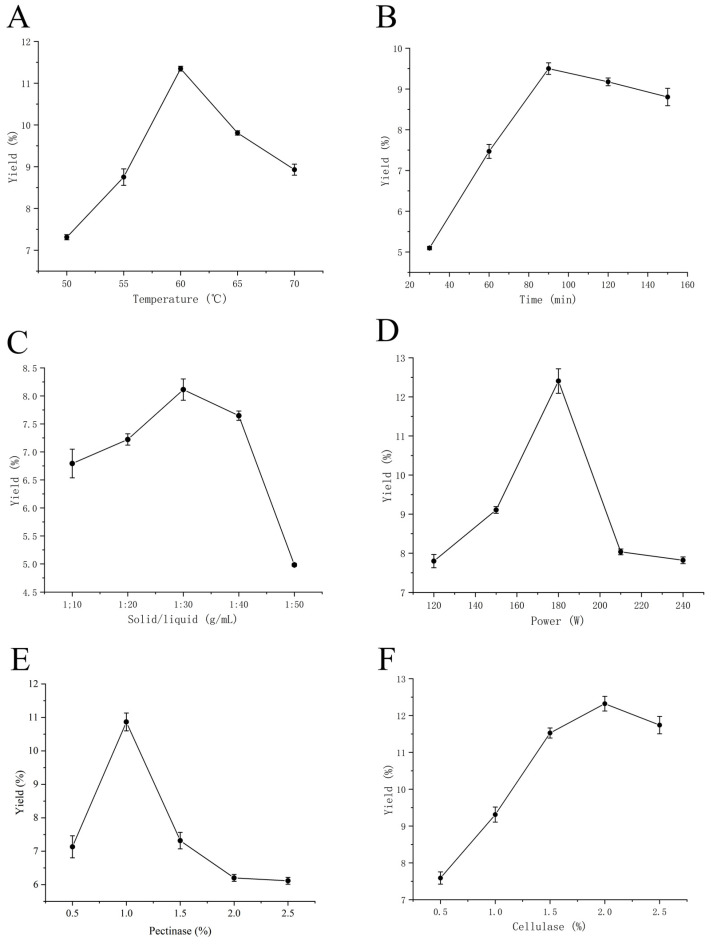
Effects of temperature (**A**), time (**B**), solid/liquid (**C**), power (**D**), pectinase (**E**), cellulase (**F**) on the yields of PSP.

As shown in Figure 1A, the polysaccharide extraction yield increased with rising extraction temperature, reaching a maximum at 60 °C before gradually declining. This may be attributed to reduced enzyme activity and structural degradation of polysaccharides at excessively high temperatures [34]. Figure 1B shows that the extraction yield increased with increasing extraction time up to 90 min, reaching a maximum (9.5 ± 0.1425%) at 90 min before declining. This decline was likely attributed to polysaccharide decomposition, degradation, or hydrolysis caused by prolonged extraction time [35]. Figure 1C indicates that extraction yield increases with increasing solvent volume (solid-to-liquid ratio from 1:10 to 1:30), peaking at 1:30 before declining. This may occur because insufficient contact between raw material and extraction solution at lower solvent volumes hinders polysaccharide dissolution. Although increasing the solvent volume enhanced polysaccharide release, excessively high solvent volumes may also dissolve other cellular components, thereby reducing polysaccharide yield [36]. As shown in Figure 1D, polysaccharide extraction yield reached its maximum (12.405 ± 0.314%) at 180 W ultrasonic power. Further increases in power led to decreased extraction yield, likely due to localized temperature increases at excessively high power levels that cause enzyme inactivation and structural damage to polysaccharides [37]. Figure 1E,F indicate that extraction yield first increases and then decreases with increasing enzyme dosage. This trend suggests that once the enzyme concentration reaches a certain level, the reaction between enzyme and substrate becomes saturated. Further increase in enzyme dosage may lead to enzyme–enzyme interactions, thereby reducing extraction yield [38]. Based on these results, ultrasonic power, extraction temperature, cellulase dosage, and pectinase dosage were selected for subsequent response surface optimization experiments.

#### 3.1.2. Extraction Optimization of Polysaccharides

The response surface experiment was designed using Design-Expert 12.0 software. The four factors with their levels and codes are listed in Table 1, the experimental design and results in Table 2, and the analysis of variance (ANOVA) results in Table 3. Regression analysis of the 29 experimental runs yielded the following equation:

Y = 12.18 + 0.4555 A + 0.1322 B + 0.5865 C − 0.0148 D + 0.0665 AB + 0.1362 AC − 0.0938 AD + 0.3292 BC + 0.4647 BD + 0.0800 CD − 2.09 A^2^ − 2.28 B^2^ − 0.6865 C^2^ − 1.62 D^2^

The model was significant (*p* < 0.0001), with a non-significant lack of fit (*p* = 0.2699 > 0.05). The *R*^2^, adjusted *R*^2^, and predicted *R*^2^ were 0.9736, 0.9471, and 0.8665, respectively, indicating that the model showed a good fit and satisfactory predictive ability.

**Figure 2:**
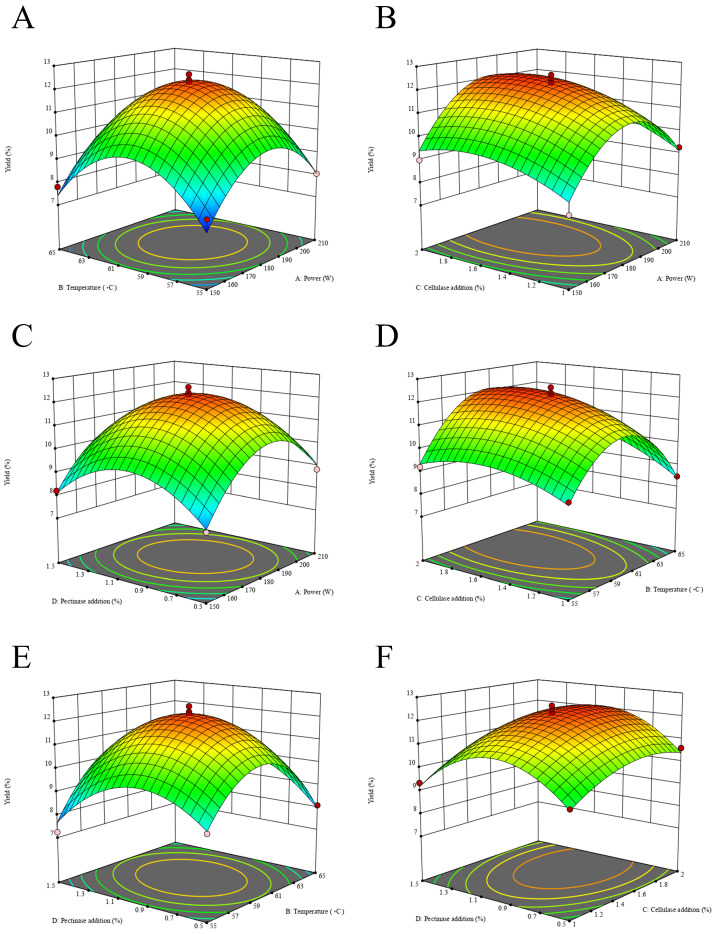
The 3D response surface plots reveal significant interactions between PSP yield and the following factors: power and temperature (**A**), power and cellulase (**B**), power and pectinase (**C**), temperature and cellulase (**D**), temperature and pectinase (**E**), and cellulase and pectinase (**F**).

Based on F-values, the factors affecting extraction yield ranked as: cellulase dosage (C) > ultrasonic power (A) > temperature (B) > pectinase dosage (D). Cellulase dosage (*F* = 31.94, *p* < 0.0001) and ultrasonic power (*F* = 19.26, *p* = 0.0006) showed extremely significant effects. Interaction analysis revealed that BD (temperature × pectinase dosage) was significant (*F* = 6.68, *p* = 0.0216), while BC (temperature × cellulase) approached significance (*F* = 3.35, *p* = 0.0884), which was consistent with the response surface plots (Figure 2). All quadratic terms were significant (*p* < 0.01), confirming the presence of nonlinear relationships between the factors and the response.

The optimal conditions predicted by the model were ultrasonic power 184 W, temperature 60.3 °C, cellulase 1.73%, and pectinase 1.01%, with a predicted yield of 12.342%. Validation under the adjusted actual conditions (ultrasonic power of 180 W, extraction temperature of 60 °C, cellulase dosage of 1.7%, pectinase dosage of 1.0%, extraction time of 90 min, and solid-to-liquid ratio of 1:30) gave an average yield of 12.45 ± 0.09%, which agreed well with the predicted value, confirming the reliability of the model.

#### 3.1.3. In Vitro Antioxidant Activity of Crude Polysaccharides

**Figure 3:**
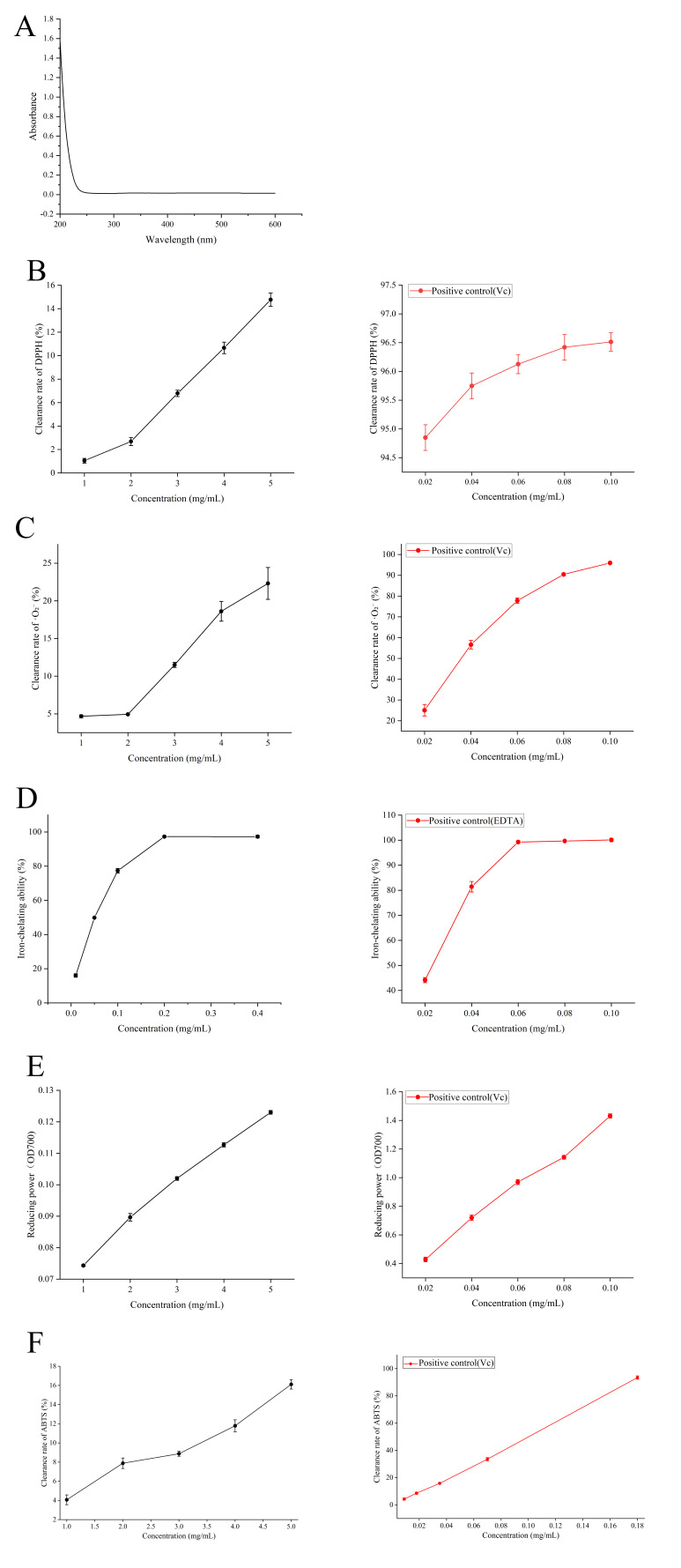
In vitro antioxidant activity results for crude polysaccharides. (**A**) UV scanning spectrum of PSP after protein removal at 200–600 nm. (**B**) DPPH radical scavenging activity of PSP and positive control Vc. (**C**) Superoxide anion radical (O^2−^) scavenging capacity of PSP and positive control Vc. (**D**) Metal ion chelating capacity (Fe^2+^) of PSP and positive control EDTA. (**E**) Reducing power of PSP and positive control Vc. (**F**) ABTS radical scavenging capacity of PSP and positive control Vc.

After protein removal using the TCA method, crude polysaccharides were subjected to UV scanning over the range of 200–600 nm. As shown in Figure 3A, no absorption peak was observed at 280 nm, indicating effective removal of proteins. The in vitro antioxidant activities of the crude polysaccharides are presented in Figure 3. As shown in Figure 3B,C, the crude polysaccharide PSP exhibited scavenging activity against both DPPH and superoxide anion radicals in a concentration-dependent manner. However, its scavenging ability was significantly lower than that of the positive control, Vc. Figure 3D shows that PSP exhibited strong ferrous ion chelating ability, reaching 97.26 ± 0.77% at 0.4 mg/mL, which approached that of the positive control EDTA. As shown in Figure 3E, the reducing power of PSP increased with increasing concentration but remained markedly lower than that of Vc. As shown in Figure 3F, PSP exhibited a dose-dependent scavenging capacity against ABTS radicals, reaching 16.11 ± 0.49% at 5 mg/mL. Overall, the results indicate that the crude polysaccharides possessed antioxidant activity, particularly ferrous ion-chelating activity. Previous studies have suggested that this strong chelating ability may be closely related to the molecular weight and high galactose content of *Pyropia suborbiculata* polysaccharide, which is consistent with the findings of Solomon et al. [26].

### 3.2. Purification of Polysaccharides

#### 3.2.1. DEAE Sepharose Fast Flow Anion Exchange Chromatography

**Figure 4:**
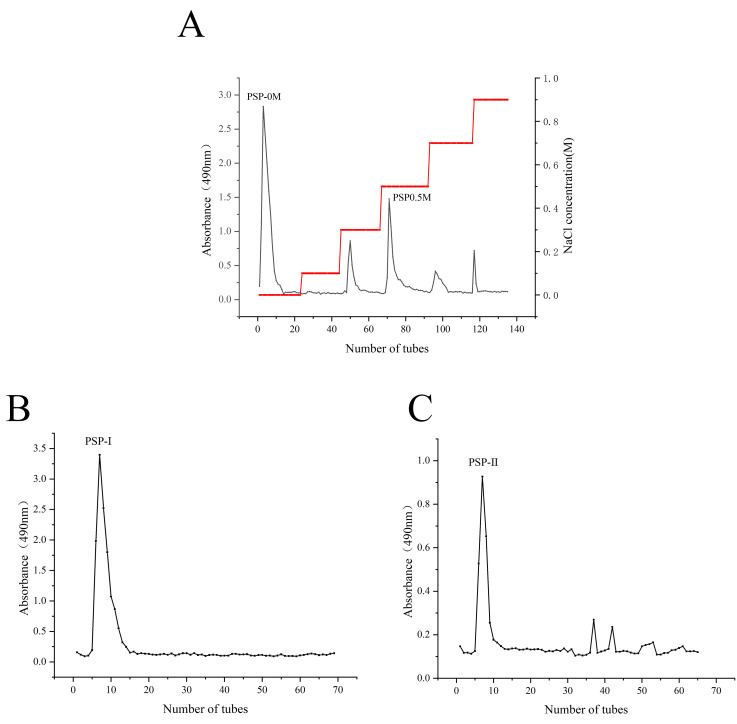
(**A**) DEAE Sepharose Fast Flow anion exchange chromatography elution curve. Two main components, PSP-0M and PSP-0.5M, were eluted. (**B**) Dextran gel chromatography elution curve of 0 M elution fraction, obtained the main component PSP-I. (**C**) Dextran gel chromatography elution curve of 0.5 M elution fraction, obtained the main component PSP-II.

The elution profile obtained from DEAE Sepharose Fast Flow anion exchange chromatography is shown in Figure 4A. Substantial amounts of purple laver polysaccharide fractions were eluted when distilled water (0 mol/L NaCl) and 0.5 mol/L NaCl were used as eluents. These fractions were collected and designated as PSP-0M and PSP-0.5M, respectively, for further purification. In contrast, only trace amounts of polysaccharides were eluted at other NaCl concentrations (OD490 < 1). Considering the large quantity of polysaccharides required for subsequent experiments, these fractions were not collected.

#### 3.2.2. Sephadex G-75 Gel Chromatography

The purification profiles of PSP-0M and PSP-0.5M obtained by Sephadex G-75 gel chromatography are shown in Figure 4B and Figure 4C, respectively. PSP-0M exhibited a single, symmetrical elution peak, indicating a relatively homogeneous molecular weight distribution. In contrast, PSP-0.5M showed a broader and less uniform elution profile, suggesting molecular weight heterogeneity. Fractions corresponding to the major peaks were collected, dialyzed, freeze-dried, and designated as PSP-I and PSP-II, respectively. Preliminary experiments showed that PSP-I exhibited superior UV damage repair activity and lower cytotoxicity. Purification results suggested relatively higher purity. Subsequent studies focused on the structural characterization and bioactivity evaluation of PSP-I.

### 3.3. Compositional Characterization of Polysaccharides

#### 3.3.1. Molecular Weight Distribution

**Figure 5:**
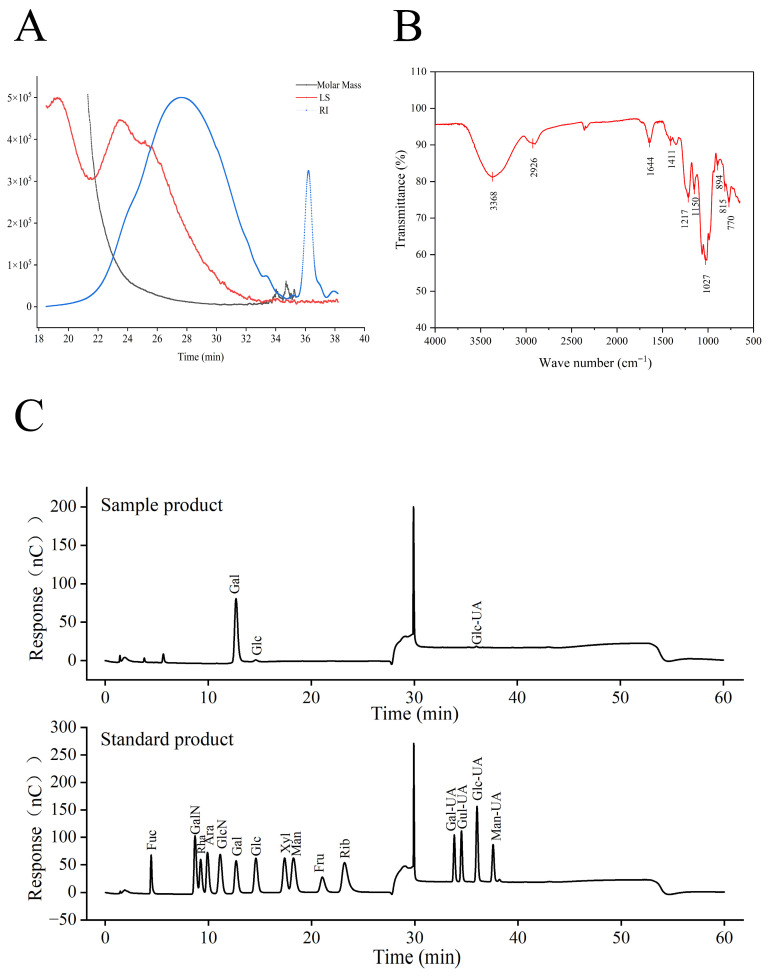
(**A**) Molecular weight distribution of PSP-I. (**B**) FT-IR spectrum of PSP-I. (**C**) Ion chromatographic curve of standards and monosaccharide composition ion chromatogram of PSP-I.

The molecular weight of PSP-I was determined by size-exclusion chromatography coupled with refractive index and multi-angle laser light scattering detection (SEC–RI–MALS). This technique enables online, real-time determination of absolute molecular weight at each elution volume by directly measuring the intensity of scattered light, thereby avoiding reliance on calibration standards required in conventional single-detector SEC methods. As a result, more accurate and reliable molecular weight information can be obtained (Figure 5A). Data analysis using ASTRA 6.1 software showed that PSP-I possessed a weight-average molecular weight (Mw) of 26.149 kDa and a number-average molecular weight (Mn) of 11.267 kDa, with a polydispersity index (Mw/Mn) of 2.321. These results indicate that PSP-I is a polydisperse system, which is typical for naturally derived polysaccharides. Within the main elution window (25–35 min), the RI detector displayed a single broad peak, suggesting that PSP-I predominantly eluted as one major component with a relatively uniform molecular weight, albeit with a certain distribution range. In contrast, weak RI signals observed at retention times beyond 35 min exhibited negligible light-scattering responses and were therefore attributed to trace low-molecular-weight species or system background noise rather than polysaccharide fractions [39].

#### 3.3.2. Monosaccharide Composition

Monosaccharide composition analysis (Figure 5C) showed that PSP-I was mainly composed of galactose (Gal), glucose (Glc), and glucuronic acid (GlcA), with a molar ratio of 96.76:2.31:0.93. These results suggest that PSP-I is a typical galactose-rich polysaccharide, consistent with the structural characteristics of polysaccharides derived from red algae. The relatively low Glc content suggests limited contamination by non-galactose polysaccharides, reflecting effective purification. The incorporation of glucuronic acid residues may contribute to the charge properties of PSP-I, which may in turn influence its solubility and potential biological activity. The monosaccharide composition of algae polysaccharides may be influenced by many factors, such as growing environment, extraction method, and degradation conditions [40].

#### 3.3.3. Fourier Transform Infrared Spectroscopy (FT-IR)

The FT-IR spectrum of PSP-I is presented in Figure 5B and exhibited characteristic absorption bands typical of macroalgal polysaccharides. A broad and intense absorption band at 3368 cm^−1^ corresponds to O–H stretching vibrations of abundant hydroxyl groups, while the band at 2926 cm^−1^ is attributed to C–H stretching vibrations [41]. The absorption peak at 1644 cm^−1^ is mainly associated with the bending vibration of bound water (H–O–H), which may overlap with the asymmetric stretching vibration of carboxylate groups (COO^−^). The peak at 1411 cm^−1^ corresponds to the symmetric stretching vibration of COO^−^, supporting the presence of glucuronic acid residues [42]. Peaks observed at 1217, 1150, and 1027 cm^−1^ fall within the polysaccharide fingerprint region and are primarily attributed to C–O–C (glycosidic linkage) and C–O stretching vibrations [43]. The band at 1217 cm^−1^ may also include contributions from sulfate ester S=O stretching vibrations, suggesting a typical polysaccharide backbone structure. In addition, the absorption peak at 894 cm^−1^ is characteristic of β-glycosidic linkages, suggesting that PSP-I predominantly contains β-type glycosidic bonds [42].

#### 3.3.4. Scanning Electron Microscopy (SEM) Analysis

**Figure 6:**
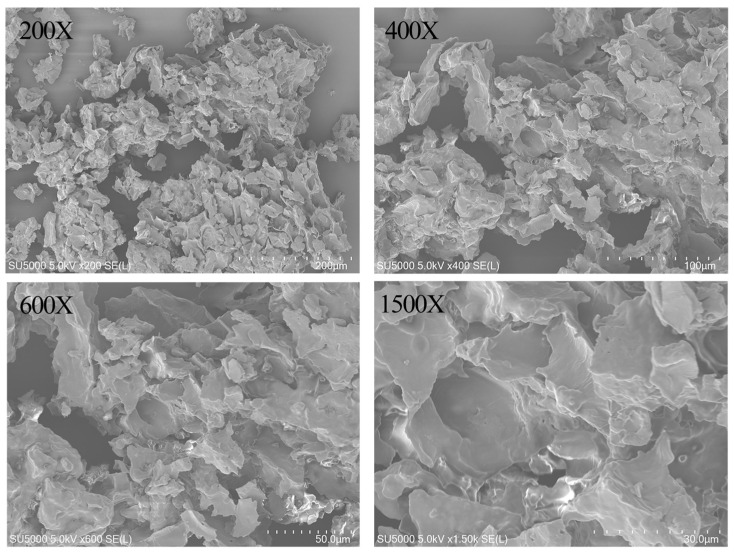
SEM images of PSP-I at magnifications of 200×, 400×, 600×, 1500×.

To further investigate the microstructural features of PSP-I, SEM was performed (Figure 6). The images show that PSP-I exhibited a typical biopolymer aggregate morphology with a porous lamellar structure and an uneven surface. At low magnifications (×200 and ×400), PSP-I appears as irregular, loosely packed aggregates composed of fragmented and thin lamellar sheets, which may be attributed to the physical exfoliation effect induced by ultrasonic treatment. At higher magnifications (×600 and ×1500), pronounced surface wrinkles, fractured edges, and a rough porous structure were observed. These features may be attributed to polysaccharide chain degradation and loosening caused by ultrasonic cavitation and enzymatic action [44]. Such morphological characteristics are generally considered to be associated with improved adsorption capacity, encapsulation ability, and bioavailability [23].

### 3.4. Protective Activity of PSP-I Against UVB-Induced Damage

#### 3.4.1. Selection of PSP-I Concentration and UVB Irradiation Dose

**Figure 7:**
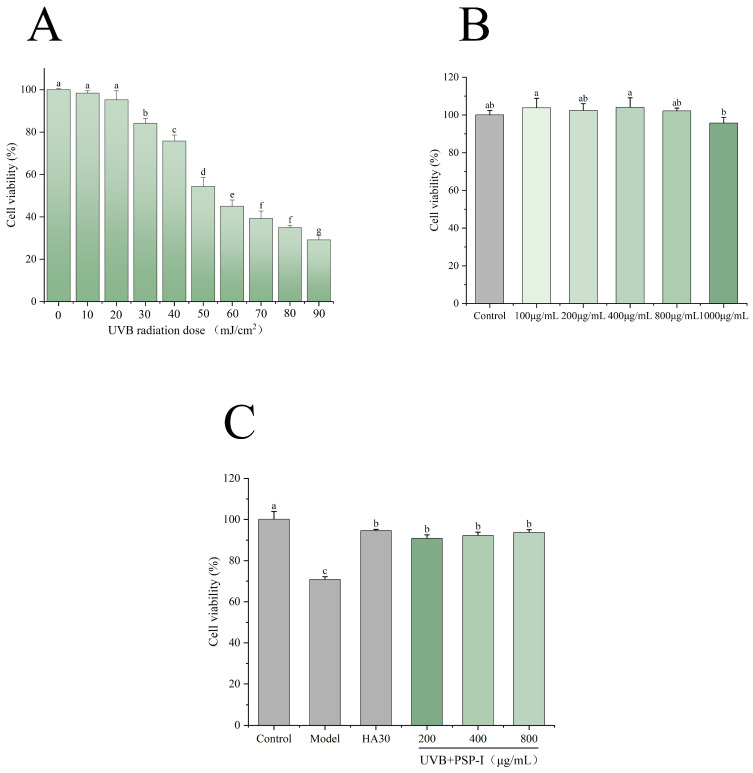
Cytotoxicity of PSP-I (**A**), UVB radiation (**B**), and the protective effect of PSP-I on UVB-damaged HaCaT cells (**C**). Data are represented as means ± standard deviations (*n* = 3). Different letters represented significant differences (*p* < 0.05).

The cytotoxicity of PSP-I at different concentrations (1000, 800, 400, 200, and 100 μg/mL) was evaluated using the CCK-8 assay. In addition, the appropriate UVB irradiation dose for establishing the photoaging model was determined by exposing HaCaT cells to various doses (0–90 mJ/cm^2^). As shown in Figure 7B, PSP-I exhibited no significant cytotoxicity within the concentration range of 100–800 μg/mL, with no statistically significant differences observed among the concentration groups compared to the control (*p* > 0.05). Therefore, concentrations of 200, 400, and 800 μg/mL were selected as the low, medium, and high treatment doses for subsequent cellular experiments. As shown in Figure 7A, cell viability decreased in a dose-dependent manner with increasing UVB radiation dose. Based on these results, a UVB radiation dose of 40 mJ/cm^2^ was selected for establishing the cellular photoaging damage model.

#### 3.4.2. Effect of PSP-I on the Viability of UVB-Damaged Cells

UVB irradiation induces photoaging-related damage in HaCaT cells, resulting in reduced cell viability. As shown by the CCK-8 assay results (Figure 7C), UVB irradiation significantly decreased HaCaT cell viability to 70.94 ± 1.28% compared with the control group (*p* < 0.05). However, treatment with PSP-I significantly reversed this effect (*p* < 0.05). At 800 μg/mL, cell viability reached 93.81 ± 1.23%, which was comparable to the 94.59 ± 0.65% observed with HA30.

#### 3.4.3. Effect of PSP-I on ROS Levels and SOD and GSH-Px Activities in HaCaT Cells

**Figure 8:**
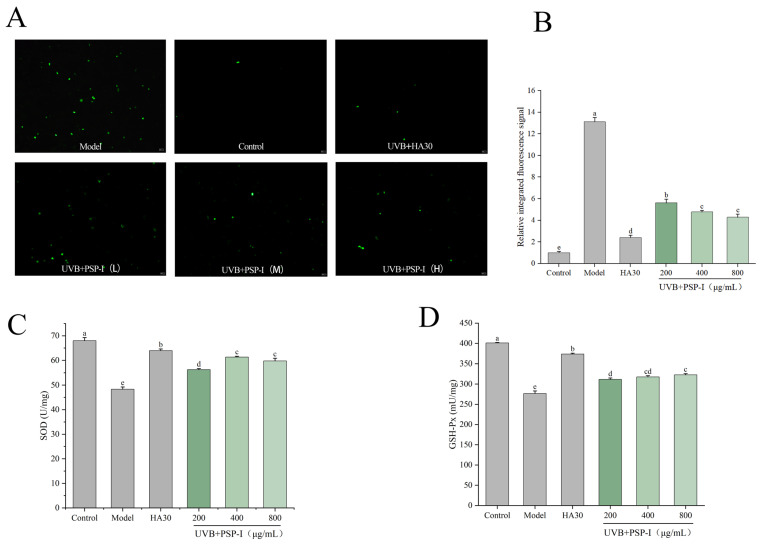
PSP-I inhibits UVB-induced intracellular ROS production, (**A**) fluorescence images of DCFH-DA observed under fluorescence microscopy. Scale bar = 250 μm. (**B**) Quantification of relative fluorescence intensity. (**C**) Effect of PSP-I on SOD activity in HaCaT cells. (**D**) Effect of PSP-I on GSH-Px activity in HaCaT cells. Data are represented as means ± standard deviations (*n* = 3). Different letters represented significant differences (*p* < 0.05).

Intracellular ROS levels were measured using the fluorescent probe DCFH-DA (Figure 8A,B). Following UVB irradiation, ROS levels in cells increased significantly. Compared with the control group, the model group exhibited an approximately 13-fold increase in intracellular ROS levels after UVB exposure. Treatment with PSP-I significantly reduced intracellular ROS to lower levels (*p* < 0.05), demonstrating a degree of concentration dependence. At the highest concentration tested (800 μg/mL), the reduction in ROS levels approached that of the positive control.

SOD and GSH-Px are important intracellular antioxidant enzymes involved in the cellular defense against UVB-induced oxidative stress [45]. Measuring their activity allows for the assessment of oxidative stress levels, thereby evaluating the anti-photoaging effects of polysaccharides. Figure 8C shows that, compared with the control group, SOD activity in the UVB model group decreased to 48.3 ± 0.92 U/mg (*p* < 0.05). Following incubation with different concentrations of PSP-I prior to UVB exposure, the UVB-induced decline in SOD activity within HaCaT cells was significantly attenuated (*p* < 0.05). At 400 μg/mL, SOD activity reached 61.32 ± 0.35 U/mg. Regarding GSH-Px activity, Figure 8D shows that UVB damage reduced GSH-Px activity to 276.63 ± 6.41 mU/mg. Pre-incubation with PSP-I significantly mitigated this decrease in a concentration-dependent manner (*p* < 0.05).

#### 3.4.4. Effect of PSP-I on Apoptosis in HaCaT Cells

**Figure 9:**
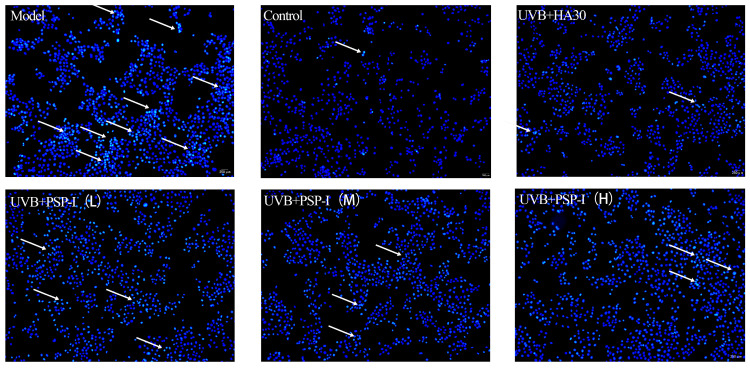
The Effect of PSP-I on UVB-Induced Apoptotic Body Formation. Fluorescent image of Hoechst 33342 observed under a fluorescence microscope. Scale bar = 250 micrometers. White arrows indicate areas of cells exhibiting distinct apoptotic features.

The protective effect of PSP-I against UVB-induced apoptosis in HaCaT cells was evaluated using Hoechst 33342 staining and fluorescence microscopy. Hoechst 33342 can permeate intact and apoptotic cell membranes and binds to the minor groove of DNA. Cells with uniform nuclear staining are considered viable, whereas chromatin condensation and/or nuclear fragmentation, accompanied by enhanced fluorescence intensity, are indicative of apoptosis [46]. As shown in Figure 9, a marked increase in apoptotic bodies was observed in the UVB model group compared with the control group. In contrast, treatment with PSP-I significantly reduced the formation of apoptotic bodies, indicating an inhibitory effect on UVB-induced apoptosis. These results suggested that PSP-I may alleviate UVB-induced photodamage by suppressing apoptosis in HaCaT cells.

#### 3.4.5. Effect of PSP-I on MMP Expression

**Figure 10:**
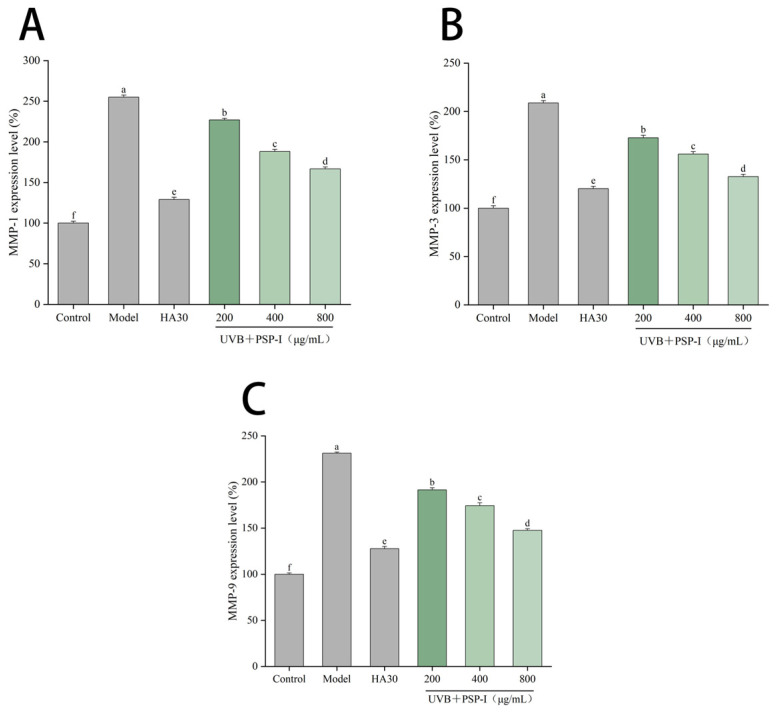
PSP-I effects on MMP-1 (**A**), MMP-3 (**B**), MMP-9 (**C**) levels in HaCaT cells. Data are represented as means ± standard deviations (*n* = 3). Different letters represented significant differences (*p* < 0.05).

The levels of MMP-1, MMP-3, and MMP-9 in the culture supernatant were measured by ELISA. MMPs are a family of collagen-degrading enzymes involved in extracellular matrix remodeling. As shown in Figure 10, with MMP levels in the control group normalized to 100%, UVB irradiation significantly increased the levels of MMP-1, MMP-3, and MMP-9 in HaCaT cells (*p* < 0.05). Treatment with PSP-I significantly reduced the levels of these MMPs in UVB-irradiated HaCaT cells in a concentration-dependent manner. These results indicate that PSP-I may exert a protective effect against UVB-induced collagen degradation by reducing MMP levels.

## 4. Discussion

*Porphyra suborbiculata* is a eurythermal and relatively rare laver species, rich in various nutrients and bioactive compounds. Research indicates that round laver exhibits relatively strong heat tolerance, a characteristic that endows it with significant potential for commercial application. However, its unique chemical composition and biological activity have yet to be systematically elucidated.

This study optimized the polysaccharide extraction from the PS-M4, which exhibits strong heat tolerance, through single-factor experiments and response surface methodology. The results show that under the optimal conditions, the polysaccharide yield reached 12.45 ± 0.09%. The purified polysaccharide PSP-I was obtained by DEAE anion exchange chromatography and Sephadex G-75 gel chromatography.

The ultrasound-assisted composite enzyme method represents an advanced extraction strategy that integrates a physical energy field (ultrasonication) with biocatalytic action (composite enzymes). Unlike conventional extraction approaches such as hot water extraction or acid/alkali extraction, the advantages of this method are not simply additive but arise from a pronounced synergistic effect between ultrasound-induced physical disruption and enzymatic hydrolysis, resulting in a substantially enhanced polysaccharide yield [47]. Bilal Muhammad Khan et al. [48] employed a hot water extraction method to isolate polysaccharides from *Porphyra haitanensis*, achieving an extraction yield of 3.3%. Shogo Isaka et al. [49] utilized a water extraction followed by alcohol precipitation method to extract polysaccharides from *Porphyra yezoensis*, yielding an extraction yield of 10.6%. Junchi Li et al. employed ultrasound-assisted optimization to extract polysaccharides from *Ginkgo biloba* leaves, achieving a maximum extraction yield of 5.37% [50]. All of these yields were lower than the 12.45 ± 0.09% obtained in this study. The purification of polysaccharides is fundamental to determining their structure. Polysaccharide purification is essential for subsequent structural characterization and bioactivity evaluation. In this study, an automated purification system was employed, which may offer advantages over traditional gravity column purification in terms of efficiency, resolution, reproducibility, and controllability.

One of the central mechanisms underlying ultraviolet B (UVB)-induced skin photoaging is the excessive generation of ROS, which subsequently triggers a cascade of cellular damage responses [51]. This oxidative stress disrupts intracellular redox homeostasis and damages macromolecules such as proteins and lipids. Moreover, elevated ROS levels act as key signaling mediators that activate the p38 and JNK signaling pathways, leading to mitochondrial release of cytochrome c and subsequent activation of the caspase cascade (caspase-9, followed by caspase-3), ultimately triggering intrinsic apoptosis [52,53,54,55]. Concurrently, ROS markedly upregulate the expression and activity of MMPs, particularly collagenase MMP-1 and gelatinase MMP-9, via activation of upstream transcription factors such as AP-1, ultimately contributing to UVB-induced skin photoaging [56,57]. Therefore, suppression of ROS accumulation, apoptosis, and collagen degradation represents an effective strategy for alleviating UVB-induced photoaging.

The results of this study suggest that PSP-I may reduce ROS production and restore antioxidant enzyme activity, thereby contributing to the regulation of oxidative stress. Decreased ROS levels may further suppress activation of the p38/JNK pathway, which could result in reduced pro-apoptotic signaling. Additionally, PSP-I may help mitigate collagen degradation by attenuating oxidative stress-induced AP-1 activation and downregulating MMP expression.

Based on the preliminary structural characterization, the anti-UVB activity of PSP-I may arise from the combined effects of several structural features, including its acidic nature, galactose-dominated backbone, moderate molecular weight, and loose, porous microstructure. These characteristics may contribute to the alleviation of oxidative stress, which could indirectly prevent UVB-induced apoptosis and collagen degradation.

The strong metal chelating capacity of PSP-I observed in this study is consistent with our previous findings and may be attributed to the presence of uronic acids. Monosaccharide composition analysis showed that PSP-I is a typical galactan polysaccharide, consistent with the structural characteristics of algal polysaccharides [58]. The high galactose content may contribute to its good water solubility and biocompatibility. It may also be associated with the strong antioxidant activity of PSP-I [59,60]. This structural characteristic may therefore underlie the metal chelating and free radical scavenging capacities of PSP-I demonstrated in this study. Molecular weight is another key factor affecting polysaccharide activity. PSP-I has a weight-average molecular weight of 26.149 kDa, within the medium range commonly observed for algal polysaccharides. This range may avoid cellular interaction limitations seen in high molecular weight polysaccharides while maintaining structural stability compared with low molecular weight components, which may enhance its anti-photoaging effects. Scanning electron microscopy revealed that PSP-I has a loose, porous lamellar structure, likely resulting from cell wall disruption during ultrasonic-assisted enzymatic extraction. This porous morphology may allow the exposure of active functional groups, which may increase their interaction with cellular targets and could enhance anti-photoaging effects [61,62]. However, further studies using techniques such as NMR and methylation analysis are needed to fully elucidate the structure–activity relationships of PSP-I.

Compared with polysaccharides from other *Porphyra* species, PSP-I exhibits three notable characteristics. First, its molecular weight (26.149 kDa) is significantly lower than that of native *P. haitanensis* (~500 kDa, Wu et al. [63]) and *P. yezoensis* (39.8–576 kDa, Huang et al. [64]) polysaccharides, and this may be attributed to ultrasound-induced chain degradation during extraction, The antioxidant capacity of PSP-I may be related to its low molecular weight (Zhou et al. [65]). Second, its galactose content (96.76%) is considerably higher than that reported for *P. haitanensis* (≈55%, Wu et al. [63]) and *P. yezoensis* (82.38–89.76%, Ji et al. [66]; Teng et al. [67]), a feature that may contribute to its strong antioxidant and anti-UVB activities. Third, while most studies on *Porphyra* polysaccharides to date have focused on antioxidant, immunomodulatory, and hypoglycemic activities (Wang et al. [68]; Teng et al. [67]), the present study provides preliminary evidence for the anti-UVB photoprotective activity of the polysaccharide from *P. suborbiculata* PS-M4.

This study investigated the extraction, purification, preliminary structural features, and anti-UVB activity of PSP-I. However, its fine structure requires further investigation. Specifically, the glycosidic linkage patterns, configurations of the main and side chains, and positions of uronic acids within the polysaccharide need to be clarified. Techniques such as nuclear magnetic resonance (NMR) spectroscopy and methylation analysis can provide a more detailed understanding of these structural aspects and their relationship to biological activity. Regarding biological effects, this study preliminarily demonstrates that PSP-I from *Pyropia suborbiculata* may alleviate UVB-induced photodamage, while suggesting possible mechanisms of action. However, the precise molecular pathways by which polysaccharides modulate UV-induced damage remain to be elucidated, including the identification of intracellular targets and regulatory mechanisms.

In summary, this work highlights the potential high-value utilization of algae, offers a reference for PSP-I polysaccharide research, and supports its potential application in health supplements and skincare products.

## 5. Conclusions

This study demonstrated that ultrasonic-assisted composite enzymatic extraction combined with response surface methodology can efficiently extract polysaccharides from *Porphyra suborbiculata* PS-M4, achieving a yield of 12.45 ± 0.09%. The crude polysaccharides exhibited notable in vitro antioxidant activity, particularly in Fe^2+^ chelation.

The purified fraction, PSP-I, exhibits structural features typical of laver polysaccharides, with a weight-average molecular weight (Mw) of 26.149 kDa, a number-average molecular weight (Mn) of 11.267 kDa, and a polydispersity index (Mw/Mn) of 2.321. Monosaccharide composition analysis showed that PSP-I consists primarily of galactose, with minor amounts of glucose and glucuronic acid in a molar ratio of 96.76:2.31:0.93, indicating that PSP-I is a galactose-rich acidic polysaccharide. FT-IR spectra displayed characteristic polysaccharide absorption bands, while SEM observations revealed a porous lamellar morphology, likely influenced by the extraction method.

Functional evaluation suggested that PSP-I may mitigate UVB-induced damage in HaCaT cells. The protective mechanism may involve reducing intracellular reactive oxygen species accumulation, restoring SOD and GSH-Px levels, inhibiting apoptosis, and downregulating the expression of MMP-1, MMP-3, and MMP-9 to prevent collagen degradation.

In summary, the PSP-I polysaccharide from PS-M4 shows promise as a natural bioactive polysaccharide for alleviating UVB-induced skin damage and represents a natural candidate for the development of functional skincare products.

## Figures and Tables

**Table 1:** Response-surface test factor levels and coding.

Encodings	A (Power, W)	B (Temperature, °C)	C (Cellulase, %)	D (Pectinase, %)
−1	150	55	1	0.5
0	180	60	1.5	1
1	210	65	2	1.5

**Table 2:** Box–Behnken central composite design for independent variables and the response values of *Porphyra suborbiculata* polysaccharides (PSP) yields.

Run	Power (W)	Temperature (°C)	Cellulase (%)	Pectinase (%)	Yield (%)
1	150	60	1	1	7.985
2	210	65	1.5	1	8.296
3	180	65	2	1	10.044
4	180	55	1.5	0.5	8.524
5	180	65	1.5	1.5	8.656
6	150	60	1.5	1.5	8.204
7	180	60	1	1.5	9.343
8	150	60	1.5	0.5	7.821
9	180	55	2	1	9.201
10	180	60	1.5	1	12.547
11	180	65	1.5	0.5	8.085
12	180	60	1.5	1	11.845
13	210	60	1.5	0.5	8.832
14	180	60	2	1.5	10.832
15	180	60	2	0.5	10.598
16	150	60	2	1	8.986
17	180	55	1.5	1.5	7.236
18	180	60	1.5	1	12.312
19	180	65	1	1	8.469
20	150	55	1.5	1	7.812
21	210	60	1.5	1.5	8.84
22	210	60	1	1	9.256
23	180	55	1	1	8.943
24	180	60	1.5	1	11.975
25	210	55	1.5	1	8.045
26	210	60	2	1	10.802
27	150	65	1.5	1	7.797
28	180	60	1.5	1	12.198
29	180	60	1	0.5	9.429

**Table 3:** ANOVA for response surface quadratic model analysis of variance.

Source	Sum of Squares	Mean Square	F-Value	*p*-Value	
Model	66.63	4.76	36.82	<0.0001	Significant
A-Power	2.49	2.49	19.26	0.0006	**
B-Temperature	0.2096	0.2096	1.62	0.2236	
C-Cellulase	4.13	4.13	31.94	<0.0001	**
D-Pectinase	0.0026	0.0026	0.0204	0.8884	
AB	0.0177	0.0177	0.1369	0.7170	
AC	0.0743	0.0743	0.5745	0.4610	
AD	0.0352	0.0352	0.2720	0.6101	
BC	0.4336	0.4336	3.35	0.0884	
BD	0.8064	0.8064	6.68	0.0216	*
CD	0.0256	0.0256	0.1981	0.6631	
A^2^	28.28	28.28	218.80	<0.0001	**
B^2^	33.84	33.84	261.81	<0.0001	**
C^2^	3.06	3.06	23.65	0.0003	**
D^2^	17.08	17.08	132.12	<0.0001	**
Residual	1.81	0.1293			
Lack of fit	1.50	0.1503	1.96	0.2699	not significant
Pure error	0.3066	0.0766			
Cor total	68.44				

Note: ** *p* < 0.01; * *p* < 0.05.

## Data Availability

The original contributions presented in the study are included in the article, further inquiries can be directed to the corresponding authors.
